# Comparative Study of Fire Resistance and Char Formation of Intumescent Fire-Retardant Coatings Reinforced with Three Types of Shell Bio-Fillers

**DOI:** 10.3390/polym13244333

**Published:** 2021-12-10

**Authors:** Feiyue Wang, Hui Liu, Long Yan

**Affiliations:** Institute of Disaster Prevention Science and Safety Technology, School of Civil Engineering, Central South University, Changsha 410075, China; wfyhn@163.com (F.W.); lhui0421@163.com (H.L.)

**Keywords:** intumescent fire-retardant coatings, shell bio-filler, fire resistance, char formation, synergistic effect

## Abstract

Three types of shell bio-fillers, including eggshell (CES), conch shell (CHS) and clamshell (CMS), were prepared by cleaning, ultrasonication and pulverizing processes of biowastes, and then applied to intumescent fire-retardant coatings. The effects of shell bio-fillers with different polymorphs on the fire resistance and char-forming of intumescent fire-retardant coatings were investigated by cone calorimeter test, fire protection tests, smoke density test, thermogravimetric analysis (TG), and the fire resistance and char-forming mechanism of bio-fillers in intumescent fire-retardant coatings were proposed. The results show that three kinds of bio-fillers exert an excellent synergistic effect on enhancing the fire resistance and char-forming properties of the intumescent fire-retardant coatings, while clamshell has the best synergistic efficiency among the bio-fillers. Especially, IFRC-CMS coating containing 3 wt% clamshell shows the best fire protection performance and lowest smoke production and heat release, which offers an equilibrium backside temperature of 134.6 °C at 900 s, a flame-spread rating of 14.4, and a smoke density rating value of 22.8%. The synergistic efficiency of bio-fillers in the intumescent coatings depends on the polymorphs of CaCO_3_ in bio-fillers, and aragonite CaCO_3_ shows a higher synergistic efficiency compared to calcite CaCO_3_ and the mixture of aragonite and calcite CaCO_3_. The CMS composed of aragonite shows the best synergistic effect, CHS composed of aragonite and calcite comes second, and CES composed of calcite has the weakest synergistic effect.

## 1. Introduction

Intumescent fire-retardant coatings are considered one of the most influential and economical materials for reducing fire hazards of buildings owing to their excellent fire resistance and heat-insulation performance, which usually include organic intumescent coatings and inorganic intumescent coatings and are widely used in wood, concrete, steel, etc. [1,2,3]. Intumescent fire-retardant coatings usually consist of an intumescent fire-retardant system, a binder, a synergist and additives, among which the intumescent fire-retardant system plays a crucial role in the properties of the intumescent fire-retardant coating. The intumescent fire-retardant system is mainly composed of three parts: a gas source, a carbon source, and an acid source, which interact to form an expanding char layer to block the transfer of heat and mass, protecting the substrate from permanent damage during combustion [4,5]. However, the traditional intumescent fire-retardant system composed mainly of APP, PER, and MEL still has the disadvantage of a low flame-retardant efficiency, so more efforts have focused on enhancing the fire resistance and char formation of traditional intumescent flame-retardant systems in polymeric materials [1].

Bio-fillers, as an environmentally-friendly material, are widely applied in fire-retardant coatings, plastics, and concrete to enhance the fire resistance, thermal stability, char formation, and mechanical performance of the materials owing to their excellent characteristics such as lightweight, high quantity, and affordability [6,7,8]. Shell bio-wastes including eggshell (CES), conch shell (CHS), and clamshell (CMS) are mainly comprised of calcium carbonate. Generally, calcium carbonate can be classified as amorphous, calcite, aragonite and vaterite, where amorphous is a non-crystalline state and calcite, aragonite, vaterite are crystalline states [9]. Calcite and aragonite are natural calcium carbonates, while vaterite is synthetic calcium carbonate. Numerous studies have reported the application of calcite and aragonite in polymeric materials. Zhou et al. used the CaCO_3_-containing oil sludge (OS) and carbon black (CB) to prepare EVA/OS/CB composites, and found that the addition of CaCO_3_-containing OS and CB could improve the flame retardancy and thermal stability of composites [10]. Zhang et al. studied the adsorption of calcite, aragonite and vaterite for CH_4_ and CO_2_ gases and found that aragonite has a stronger catching ability than calcite [11]. Radek et al. synthesized calcium carbonate of calcite, aragonite, and vaterite and investigated the effect of polymorphs calcium carbonate on nano-lime suspensions, and found that the addition of calcium carbonate with aragonite was effective in reducing the propagation of cracks [12]. Liu et al. used argopecten irradians and mactra veneriformis shells to remove phosphate from aqueous solutions and showed that calcium carbonate with calcite has a stronger adsorption for phosphate than calcium carbonate with aragonite [13]. Li et al. used steel fiber-polyvinyl alcohol fiber-calcium carbonate whisker multi scale fiber to reinforce cementitious composites, and the addition of calcium carbonate whisker can effectively enhance the mechanical properties of the composites [14]. In general, CES is mainly composed of calcite CaCO_3_, and CMS primarily consists of aragonite CaCO_3_, while CHS is composed of a mixture of calcite and aragonite CaCO_3_. Among them, calcite CaCO_3_ has strong thermal stability, while aragonite CaCO_3_ has superior advantages on enhancing the mechanical properties and processing properties of polymeric materials. The enhanced effect of calcium carbonate in polymeric materials varies with the polymorphs of calcium carbonate. However, few studies have reported the effect of polymorphs of calcium carbonate on the fire resistance and char-forming properties of intumescent fire-retardant coatings.

In this study, eggshell, conch shell, and clamshell are used as raw materials to prepare three types of shell bio-fillers through cleaning, ultrasonication, and pulverizing processes. Four kinds of intumescent fire-retardant coatings were prepared with APP-PER-MEL as intumescent flame retardant, shell bio-fillers as synergists, and waterborne epoxy resin as binder, and the effects of bio-filler polymorphs on the combustion properties of intumescent fire-retardant coatings were investigated. Furthermore, thermogravimetric analysis (TG), scanning electron microscopy (SEM), cone calorimeter test, Fourier transform infrared spectroscopy (FTIR), X-ray diffraction analysis (XRD) were used to analyze the synergistic mechanism of different bio-fillers in the intumescent coatings.

## 2. Experimental

### 2.1. Materials

Eggshell, clamshell, and conch shell were collected from the faculty and staff canteen of Central South University. Waterborne epoxy hardener (Ruicheng Selected New Materials Technology Co., Ltd., Guangzhou, China) used as amine curing agent is combined with waterborne epoxy resin (Ruicheng Selected New Materials Technology Co., Ltd., Guangzhou, China) as a film-forming polymer. The additives including dispersant and defoamer were obtained from Qingdao Xingguo Coatings Co., Ltd., Qingdao, China. The MEL (purity ≥ 99.5%), PER (purity: 99.5%), and APP (water solubility ≤ 0.04%, purity: 99.5%) were provided by Hangzhou JLS Flame Retardant Chemical Co., Ltd., Hangzhou, China.

### 2.2. Preparation of Materials

#### 2.2.1. Preparation of the Shell Bio-Fillers

Firstly, three kinds of shell biowastes including eggshell, conch shell, and clamshell were washed in deionized water to remove dirt, and then cleaned with 4% NaOH to remove impurities and organic matter on the surface of bio-fillers. Afterward, the samples were continually washed with deionized water until neutralization, and ultrasonicated for 2 h in an ultrasonic instrument (Kunshan Ultrasonic Instrument Co., Ltd., Kunshan, China). Thirdly, the samples were dried in 80 °C oven for 48 h. Lastly, the samples were ground in a planetary ball mill (Nanjing Chishun Science & Technology Co., Ltd., Nanjing, China), and placed in a shaker (Xinxiang Rucheng Machinery Equipment Co., Ltd., Xinxiang, China) to obtain bio-fillers with a particle size distribution of 50–80 μm. The resulted samples were named as CES, CHS, CMS, respectively.

#### 2.2.2. Preparation of Intumescent Fire-Retardant Coatings

APP, MEL, and PER with a mass ratio of 3:1:1.5 were blended to prepare intumescent fire retardant (IFR), which is mixed with bio-fillers and deionized water to prepare the coating slurry by stirring at 1000 r/min for 20 min in a high-speed disperser. Then, waterborne epoxy resin, dispersant, and defoamer were incorporated into the slurry and stirred for 20 min at 500 r/min. Finally, intumescent fire-retardant coatings were prepared by combining waterborne epoxy hardener and slurry at 500 r/min for 20 min. The prepared coatings were applied to several plywood boards of different sizes (150 mm × 150 mm × 4 mm, 75 mm × 75 mm × 4 mm, 600 mm × 90 mm × 4 mm) at a wet density of 500 g/m^2^ and several plywood boards of 300 mm × 150 mm × 4 mm at a wet density of 250 g/m^2^. The intumescent fire-retardant coatings were prepared without bio-filler and with 3 wt% bio-filler, and the specific formulation is shown in Table 1.

### 2.3. Characterization

#### 2.3.1. Fourier Transform Infrared Spectroscopy

FTIR tests used KBr pellets in an iCAN9 FTIR spectrometer (Tianjin Nengpu Technology Co., Ltd., Tianjin, China) to characterize char residues and bio-fillers.

#### 2.3.2. Scanning Electron Microscopy

SEM was applied to study the micro-morphology of the bio-fillers and char residues in the MIRA 3 LMU scanning electron microscopy (Tescan, Brno, Czech Republic) at a voltage of 20.0 kV.

#### 2.3.3. X-ray Diffraction

XRD was tested with conditions of Cu–Ka radiation, a rate of 5°/min, and a range of 5 to 70° in an Advance D8 XRD diffractometer (Bruker, Fällanden, Switzerland).

#### 2.3.4. Smoke Density Test

Smoke density test was used to test maximum light absorption rates and smoke density rating values on a PX-07-008 tester (Suzhou Phinix Instruments Co., Ltd., Suzhou, China) in accordance with the GB/T8627-2007.

#### 2.3.5. Fire Protection Tests

Fire protection properties of the samples were assessed by the big panel method, cabinet method, tunnel method tests according to GB12441-2018 standard procedure. In cabinet method tests, the weight loss (the difference of the sample mass before and after the test), char index, and intumescent factor (the ratio of the thickness of the coatings after and before test) of the samples were determined. The char index was calculated by Formula (1):

(1) $$Char\;index=\frac{\sum_{i=1}^{n}(a_{i}b_{i}h_{i})}{n}$$

where: *a_i_* is defined as the char length (cm), *b_i_* is defined as the char width (cm), *h_i_* is defined as the char depth (cm), and *n* is the number of samples.

Tunnel method test was conducted using a SDF-2-type 2-foot flame tunnel instrument (Jiangning Analysis Instrument Company, Nanjing, China). The flame spread over the coating surface of the samples was evaluated when ignited under controlled conditions in a small tunnel, and the flame-spread rating (*FSR*) of the samples was calculated by Formula (2):

(2) $$FRS=\frac{L_{s}-L_{a}}{L_{r}-L_{a}}\;$$

where: *L_s_* is the mean of five flame advance readings of samples (mm), *L_a_* is the mean of five flame advance readings of asbestos board (mm), and *L_r_* is the mean of five flame advance readings of oak board (mm).

#### 2.3.6. Cone Calorimeter Test 

The cone calorimeter test was conducted at a heat flux of 50 kW/m^2^ in the aluminum foil following ISO 5660-2002 standard procedure.

#### 2.3.7. Thermogravimetric Analysis

TG analysis was carried out at a heating rate of 10 °C/min, and the samples were heated from 35 °C to 800 °C under a nitrogen atmosphere, using a TGA/SOTA 851 thermogravimetric instrument (Mettretoli Instruments Co., Ltd., Zurich, Switzerland). The theoretical char residue (*W*_theo_) of intumescent fire-retardant coating was calculated using Formula (3):

(3) $$W_{\mathrm{theo}}\left(t\right)=\overset{n}{\underset{i=1}{\sum }}\chi_{i}W_{i}\left(t\right)\;$$

where $\chi_{i}$ is the percent of compound *i*, %; and $W_{i}\left(t\right)$ is the residual amount of char layer for compound *i* at *t* °C.

## 3. Results and Discussion

### 3.1. Morphology and Composition of Bio-Fillers

**Figure 1:**
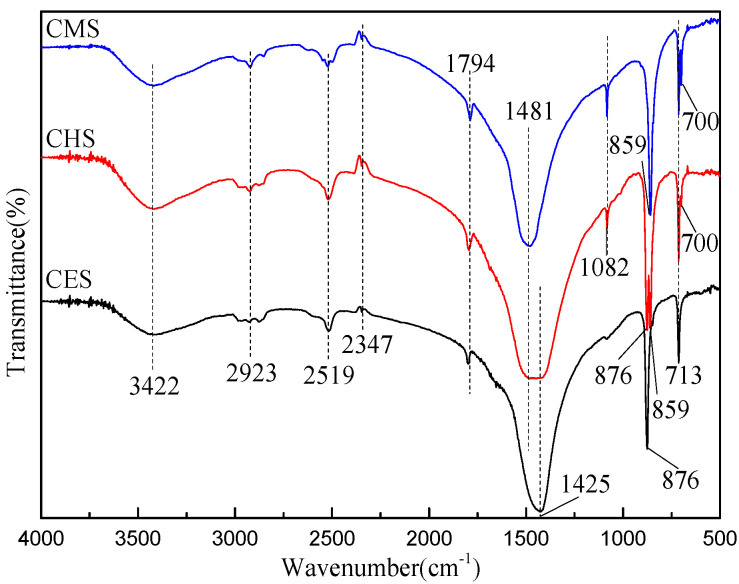
FTIR spectra of different shell bio-fillers.

The FTIR spectra of CES, CHS, and CMS are shown in Figure 1, and the FTIR assignments for the functional groups of different shell bio-fillers in Table 2. The characteristic peaks of shell bio-fillers are located at 700, 713, 859, 876, 1082, 1452, 1481, 1794, 2347, 2519, 2923, 3422 cm^−1^, while the peaks at 1481, 876, 713 cm^−1^ are associated with the forms of calcite, and the peaks at 1425, 1082, 859, 713, 700 cm^−1^ are assigned to the forms of aragonite [15]. Due to the different vibrational modes of CO_3_^2−^, calcium carbonate with polymorphs can be accurately identified from the absorption peaks at 1481–1425, 1082, 876–859, and 700–713 cm^−1^. The strong absorption peak at 1481 cm^−1^ originates from the asymmetric stretching of aragonite, while the asymmetric stretching vibration of calcite appears near 1425 cm^−1^. The C-O stretching vibration peak of aragonite is observed at 1082 cm^−1^, and the absorption peaks at 876 and 860 cm^−1^ are the out-of-plane deformation vibration peaks of CO_3_^2−^ for calcite and aragonite, respectively. The difference in the O–C–O bending frequency of aragonite and calcite is less than 2 cm^−1^, and these modes are recognized as a single absorption near 713 cm^−1^ in the spectra [16,17,18,19,20]. In conclusion, CES and CMS are composed of calcite CaCO_3_ and aragonite CaCO_3_, respectively, while CHS is a mixture of calcite and aragonite CaCO_3_.

**Figure 2:**
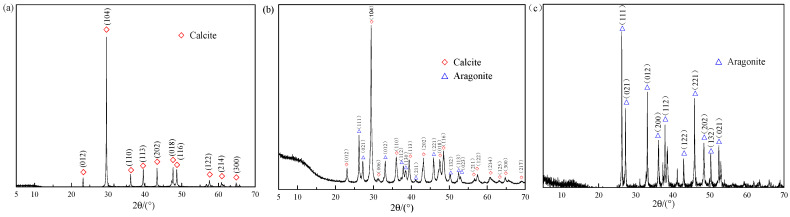
XRD patterns of shell bio-fillers. (**a**) CES; (**b**) CHS; (**c**) CMS.

The XRD patterns of the shell bio-fillers are shown in Figure 2. The XRD pattern of the CES bio-filler matches with the calcite (CaCO_3_, PDF#86-2334) pattern and the main phases of the CHS powder are identified as calcite (CaCO_3_, PDF#86-0174) and aragonite phases (CaCO_3_, PDF#75-2230). The CMS belongs to a typical aragonite structure (CaCO_3_, PDF#71-2396) without any characteristic of the calcite phase. These results are confirmed by the results of FTIR spectra in Figure 2, which is also consistent with the previous reports [7,8,19,23].

**Figure 3:**
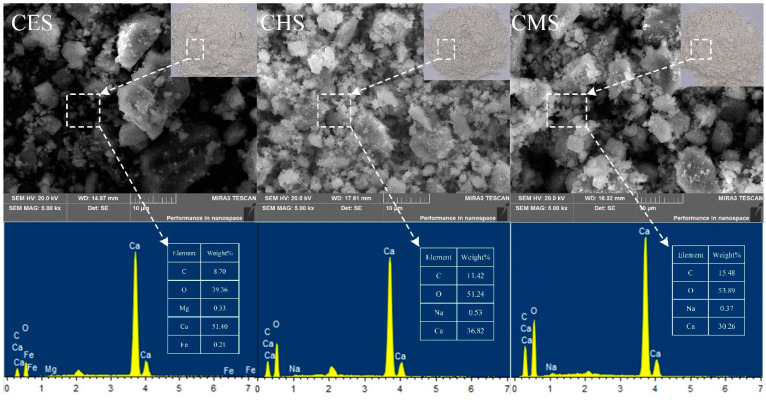
SEM images and EDS maps of shell bio-fillers.

The morphologies and compositions of CES, CHS, and CMS are presented in Figure 3. As seen in Figure 3, all the bio-fillers show an irregular blocky structure with a rough surface, and the size is mainly distributed in the range of 1–20 μm. By combining EDS results, it can be found that all the shell fillers mainly consist of Ca, O, and C elements, where there is a slight difference in content of Ca element.

**Figure 4:**
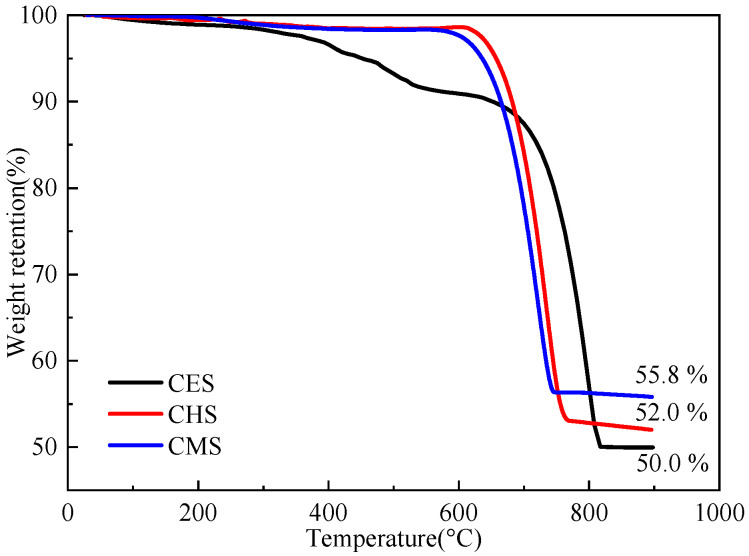
TG curves of shell bio-fillers.

The TG curves of CES, CHS, and CMS are shown in Figure 4. As observed from Figure 4, the pyrolysis process of shell bio-fillers is mainly divided in two stages in the temperature ranges of 100–550 °C and 550–900 °C. The mass loss at 150–550 °C is attributed to the decomposition of organic matter, and the mass loss at 550–900 °C is assigned to the decomposition of calcium carbonate. Specifically, CES showed a higher mass loss at 150–550 °C due to the organic matter rich in CES. The char residues of CES, CHS, and CMS samples at 900 °C are 50.0%, 52.0%, and 55.8%, respectively, where CMS exerts the highest residual weight.

### 3.2. Fire Protection Tests

The tunnel method and cabinet method tests for the intumescent fire-retardant coatings applied in wood substrates are presented in Table 3. As seen in Table 3, the addition of shell bio-fillers exerts an excellent synergistic efficiency on enhancing the fire protection performance of intumescent fire-retardant coatings, among which the CMS shows the best synergistic effect. Especially, compared to IFRC-0 without bio-filler, the IFRC-CMS sample shows a 13.5% reduction in weight loss, 35.8% reduction in char index, 31.1% reduction in flame-spread rating, and 50.0% increase in intumescent factor. The results indicate that the shell bio-fillers can effectively improve the fire resistance performance of intumescent fire-retardant coatings, and the synergistic effect of shell bio-filler depends on the polymorphs of CaCO_3_, in which the synergistic efficiency of aragonite CaCO_3_ is higher than that of calcite CaCO_3_.

**Figure 5:**
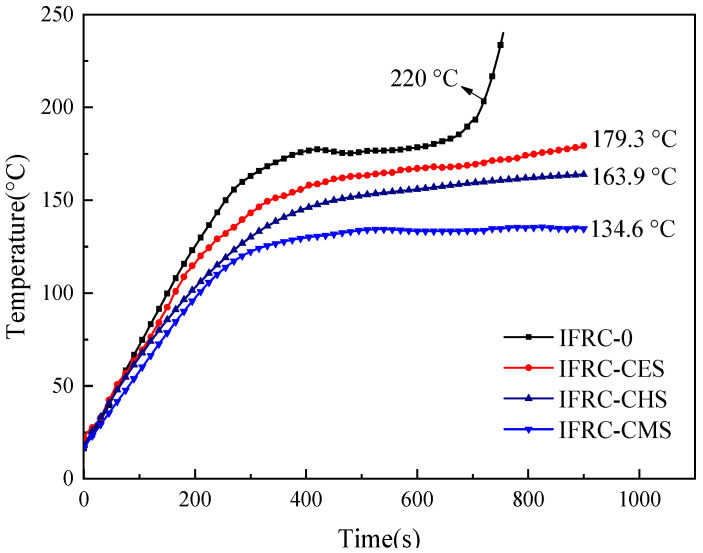
Backside temperature curves of IFRCs assessed by the big panel method test.

The results from the big panel method test for the intumescent fire-retardant coatings applied in wood substrates are illustrated in Figure 5. From Figure 5, the IFRC-0 without shell bio-filler exhibits a poor heat-insulation performance with a fire-resistant time of 700 s. The presence of shell bio-fillers induces a significant slowdown in the rate of temperature rise during combustion, where the equilibrium backside temperatures of IFRC-CES, IFRC-CHS, and IFRC-CMS are 179.3, 163.9, and 134.6 °C at 900 s, respectively. The results reveal that shell bio-filler can effectively improve the heat-insulation property of intumescent fire-retardant coatings, where IFRC-CMS with 3 wt% shell bio-filler performs the best synergistic flame-retardant effect. This is consistent with the results of the tunnel method and cabinet method tests.

**Figure 6:**
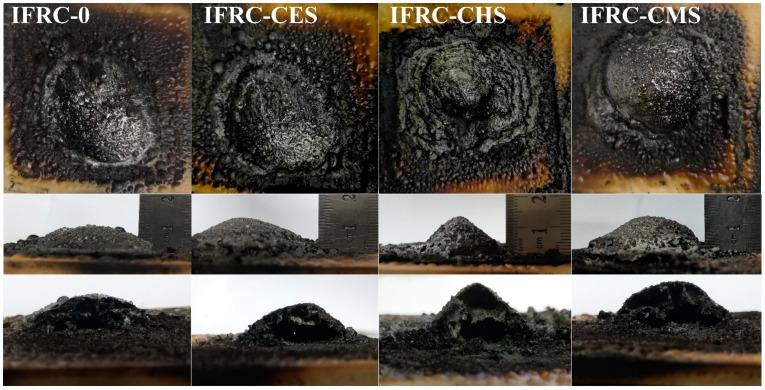
Photographs of the char residues obtained from the big panel method test.

**Figure 7:**
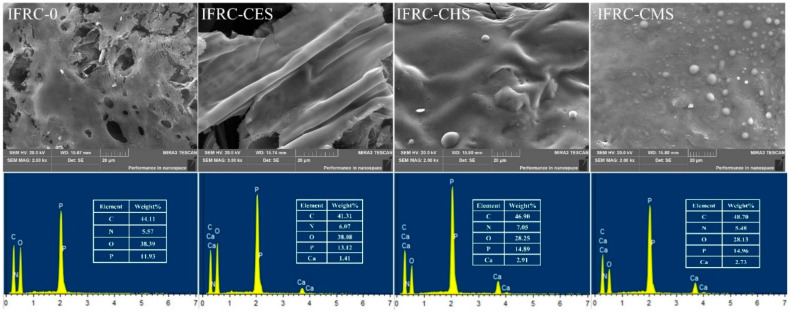
SEM images and EDS maps of the char residues obtained from the big panel method test.

To further investigate the synergistic effect of shell bio-fillers with different polymorphs on intumescent fire-retardant coatings, the morphologies of the char layer were analyzed. As shown in Figure 6, the IFRC-0 sample without shell bio-filler shows a low expansion ratio and large charring area, corresponding to a weak heat-insulation property. After addition of shell bio-fillers, the coatings form a higher intumescent char, and the char height is 11.0, 12.5, and 14.0 mm for IFRC-CES, IFRC-CHS, and IFRC-CMS, respectively. This phenomenon implies that the addition of shell bio-fillers can promote the intumescent process of the char layer to block the transfer of heat and mass, while effectively inhibiting the decomposition of the underlying materials. The IFRC-CMS exhibits the best fire resistance properties among the samples, which is attributed to the fact that calcite CaCO_3_ has stronger adsorption for phosphate than aragonite CaCO_3_ that plays a negative effect on the intumescent process of the coatings [13]. By combing the results of SEM-EDS from Figure 7, the char layer with shell bio-fillers contains higher amounts of P and Ca elements, in which the IFRC-CMS presents the highest content. This phenomenon means that the shell bio-filler with aragonite can promote the formation of more phosphorus-rich and calcium-rich cross-linked structures in the char layer that improve the strength of the char layer. With the addition of same amount of bio-filler, the CMS is prone to endow the coating with excellent expansion and char-forming ability concomitant with superior fire resistance.

### 3.3. Cone Calorimeter Test

**Figure 8:**
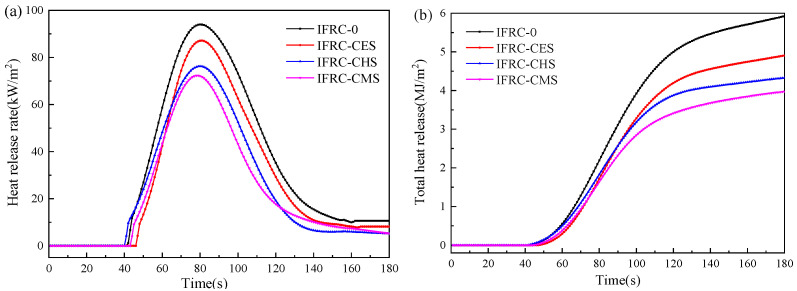
HRR (**a**) and THR (**b**) curves of IFRCs.

The cone calorimeter test is an effective way to evaluate the fire protection performance for the intumescent fire-retardant coatings applied in wood substrates, and the results are illustrated in Figure 8 and Table 4. The peak heat release rate (PHRR) value and total heat release (THR) for the IFRC-0 are 94.0 kW/m^2^, 5.9 MJ/m^2^ respectively, and the addition of shell bio-fillers effectively reduces the THR and PHRR values of the coatings. Compared with IFRC-0, the PHRR and THR values are decreased by 7.2% and 16.9% for IFRC-CES, 18.8% and 27.1% for IFRC-CHS, 23.1% and 32.2% for IFRC-CMS, respectively, indicating that all the shell bio-fillers have a good synergistic efficiency in reducing the heat release. Among the samples, IFRC-CMS sample presents the lowest PHRR and THR values. However, the synergistic efficiency of bio-filler in the intumescent fire-retardant coatings depends on the polymorphs of the shell bio-fillers, and the synergistic efficiency of bio-filler is enhanced with the increase amount of aragonite CaCO_3_ in bio-filler. This phenomenon is ascribed to the fact that the shell bio-filler can react with the phosphoric acid derivatives released from APP to form thermally stable calcium metaphosphate and calcium phosphate on the surface of the char layer that effectively block the transfer of mass and heat, thus enhancing the fire resistance [24]. Nevertheless, calcite has stronger adsorption for phosphate than aragonite to cause the reduction of acid source [13], which suppresses the dehydration of carbon source, carbonization, and chain decomposition of the intumescent fire-retardant coatings, thus diminishing the expansion and char-forming rate of the char layer as well as the synergistic flame-retarded efficiency.

### 3.4. Smoke Density Test

**Figure 9:**
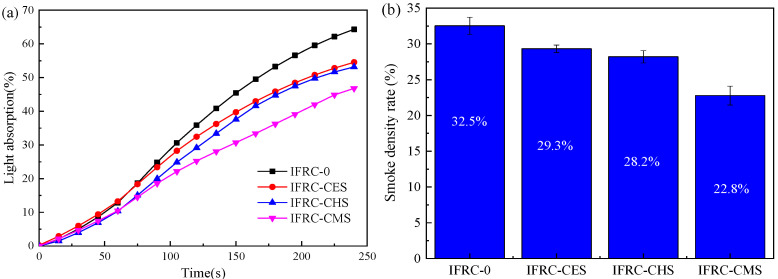
Light-absorptivity curves (**a**) and smoke density rate (**b**) of IFRCs.

The smoke density rate (SDR) and light absorption curves for the intumescent fire-retardant coatings applied in wood substrates are presented in Figure 9. The IFRC-0, IFRC-CES, IFRC-CHS, and IFRC-CMS show the maximum light absorption values of 64.3%, 54.6%, 53.2%, and 46.8%, respectively, and SDR values of 32.5%, 29.3%, 28.2%, and 22.8%, respectively. Compared to IFRC-0, the addition of shell bio-filler can effectively decrease the smoke density rating values and light absorption rate of intumescent fire-retardant coatings. IFRC-CMS sample shows the lowest smoke density rating values and light absorption rate, which is ascribed to the formation of more residual weight, as supported by TG analysis.

### 3.5. Thermal Stability Analysis

**Figure 10:**
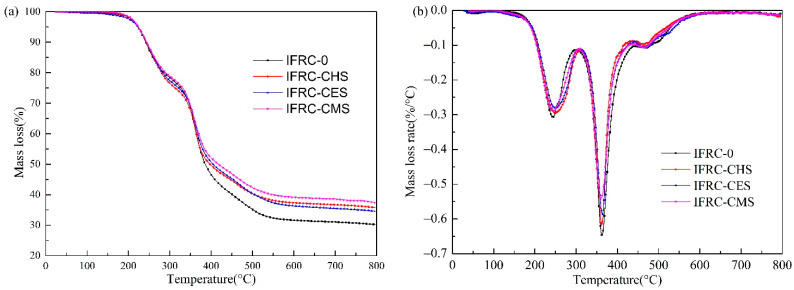
TG (**a**) and DTG (**b**) curves of IFRCs under nitrogen atmosphere.

The TG and DTG curves of the coatings are illustrated in Figure 10, and the thermal data is depicted in Table 5. From Figure 10, it can be observed that the pyrolysis process of intumescent fire-retardant coatings mainly occurs in the temperature ranges of 100–315 °C, 315–445 °C, and 445–600 °C. The first stage at 100–315 °C is mainly caused by the volatilization of small molecules from the waterborne epoxy resin and flame retardants, which results in more than 20% weight loss. The second stage at 315–445 °C is ascribed to the interaction of MEL, PER, and APP to form an expanded char layer, in which APP will decompose to produce phosphoric acid to catalyze the esterification of PER into molten char, while MEL will generate a large amount of inert gas that promote the expansion of molten char to form intumescent char, effectively blocking the transfer of mass and heat. The third stage at 445–600 °C is the reaction stage of the shell bio-filler with the phosphoric acid to form thermally stable calcium metaphosphate and calcium phosphate to enhance the thermal stability of char layer at high temperature. The IFRC-CMS sample acquires the highest residual weight of 37.4% at 800 °C.

As shown in Table 5, the addition of CES causes a decrease in the initial decomposition temperature (*T*_0_) and an increase in the temperature of peak mass loss rate (*T*_m_), while the addition of CHS and CMS leads the coating to show the opposite trends. However, there is no obvious differences in *T*_0_ and *T*_m_ caused by CES, CHS, and CMS. In addition, the experimental residual weight (*W*_exp_) of IFRC-0, IFRC-CES, IFRC-CHS, and IFRC-CMS coatings, which are 30.2%, 34.6%, 35.8%, and 37.4% respectively, are significantly higher than the corresponding theoretical residual weight (*W*_theo_). This results reveal that the existence of interaction among the waterborne epoxy resin, intumescent flame retardant and shell bio-fillers, and the IFRC-CMS has a maximum ΔW of 19.7% [24]. This phenomenon may be attributed to the fact that shell bio-fillers can react with phosphoric acid to form stable calcium metaphosphate and calcium phosphate to enhance the strength of the char layer. However, the presence of calcite CaCO_3_ will reduce the synergistic efficiency of bio-fillers due to the strong adsorption between calcite and phosphate, as supported by Liu [13]. The above results show that the presence of shell bio-filler can effectively improve the expansion and char-forming ability of intumescent fire-retardant coatings, in which CMS composed of aragonite CaCO_3_ exerts excellent synergistic efficiency.

### 3.6. Fire Resistance and Char Formation Mechanism

**Figure 11:**
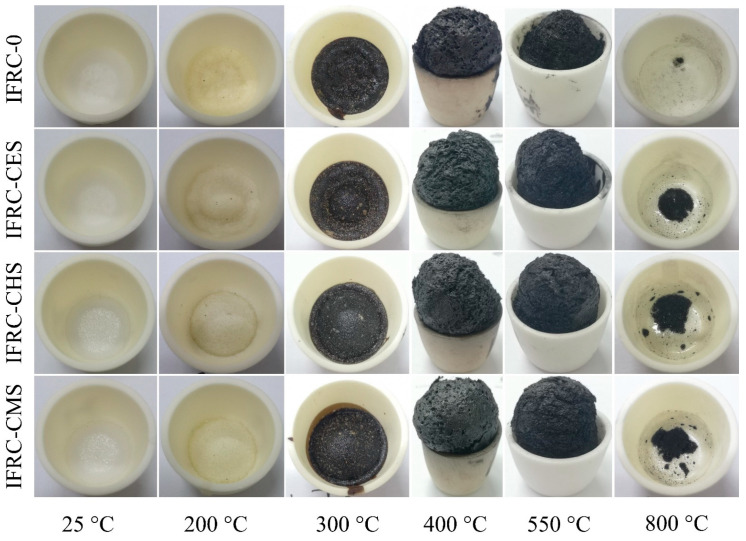
Photographs of char residues for IFRCs under different treatment temperatures.

To further investigate the synergistic fire resistance and char formation mechanism of shell bio-fillers in intumescent coatings, the coatings were heated in a muffle furnace under different temperatures, and the photographs of the char layer are presented in Figure 11. When the temperature increases to 200 °C, the coatings transfer to yellow sample and occur expansion phenomenon. When the temperature rises to 300 °C, the coatings start to carbonize, where the IFRC-CMS presents a better expansion. When the temperature reaches 400 °C, MEL PER, and APP interact to form an expanding char layer, among which IFRC-CMS has the optimal expansion and char formation. As the temperature continues to rise, the char layer begins to decompose, and IFRC-CMS coating has the highest residual weight at 800 °C.

**Figure 12:**
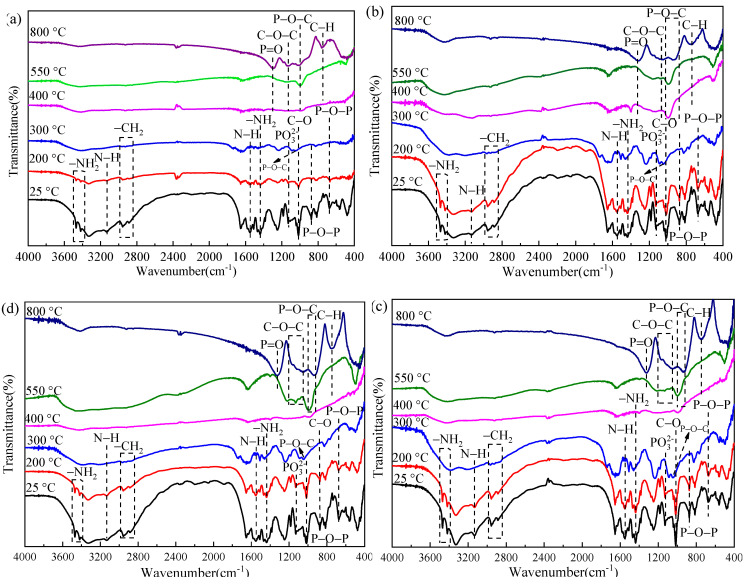
FTIR spectrum of intumescent fire-retardant Coatings under different treatment temperatures. (**a**) IFRC-0; (**b**) IFRC-CES; (**c**) IFRC-CHS; (**d**) IFRC-CMS.

Figure 12 shows the FTIR spectra of coatings under different treatment temperatures, and the assignments of characteristic groups are listed Table 6. As can be seen from Figure 12, when the temperature is up to 300 °C, the absorption peaks of –NH_2_ (3470, 3420 cm^−1^), –CH_2_ (2956, 2886 cm^−1^), N–H (1553, 3136 cm^−1^), PO_3_^2−^ (1128 cm^−1^), C–O (1016 cm^−1^), and P–O–P (874, 674 cm^−1^) functional groups disappear, and a new stretching vibrational peak appears at 1045 cm^−1^ assigned to P–O–C group [25,26,27,28,29,30,31,32,33,34], indicating that APP decomposes to phosphoric acid derivatives that catalyze PER esterification into carbon. When the temperature reaches 400 °C, the absorption peaks of the main functional groups for the original coating have basically disappeared, indicating that the coatings have degraded or engaged in the carbonization reaction to form intumescent char via the interaction of APP, PER, and MEL. As the temperature continues to increase, the formed expanded char layer starts to decompose. Compared with IFRC-0, the samples containing shell bio-fillers exhibit stronger absorption peaks of P=O (1286 cm^−1^), C–O–C (1127 cm^−1^), P–O–C (1017, 944 cm^−1^), and C–H (758, 738 cm^−1^) functional groups [35,36,37,38,39], indicating that the presence of bio-fillers can induce the formation of more aromatic structures and phosphorus-rich cross-linked structures. In general, the presence of these cross-linked and aromatic structures can improve the thermal stability of the expanded char layer, thus expressing a higher amount of char residue in Figure 11.

**Figure 13:**
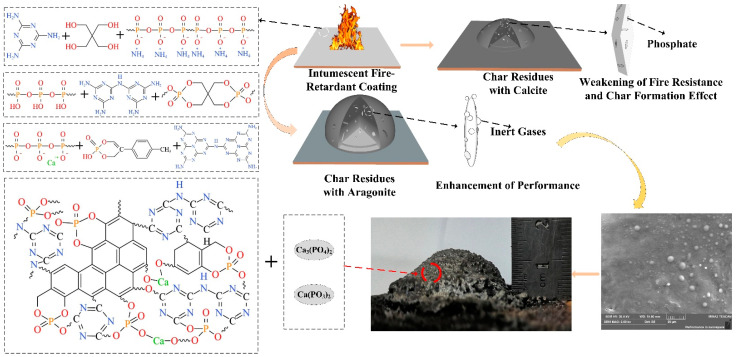
Fire resistance and char-forming mechanism of the different shell bio-fillers.

The fire resistance and char formation mechanism of shell bio-fillers in intumescent fire-retardant coatings are depicted in Figure 13. As seen in Figure 13, APP decomposes to generate phosphoric acid, metaphosphoric acid, and non-combustible gas, where phosphoric acid and metaphosphoric acid will catalyze the esterification of PER into molten char. In this stage, the bio-fillers can react with phosphoric acid and metaphosphoric acid to form stable calcium metaphosphate and calcium phosphate that enhances the strength and compactness of the char layer. With the increase of temperature, MEL will cyclize and decompose into triazine compounds concomitant with the release of a large amount of non-combustible gas that promotes the expansion of the char layer and dilutes the fuel gases to reduce the intensity of combustion. The synergistic efficiency of bio-fillers depends on the polymorphs of shell bio-fillers, and aragonite CaCO_3_ shows higher synergistic efficiency than calcite CaCO_3_, which is attributed to the fact that calcite CaCO_3_ has stronger adsorption for phosphate than aragonite CaCO_3_ that plays a negative effect on the intumescent process of the coatings. Compared to calcite CaCO_3_, aragonite CaCO_3_ has a lower mass loss to coatings, effectively reducing the amount of heat release and smoke generation. Therefore, the addition of CMS exhibits excellent synergistic efficiency in improving the fire resistance and char-forming property. The CMS shows the highest synergistic efficiency in the intumescent coating with the same content, CHS comes second, and CES shows the least effect.

## 4. Conclusions

In this paper, three kinds of shell bio-fillers were prepared from eggshell, conch shell, and clamshell, respectively, and carefully characterized by FTIR, XRD, SEM, and TG analyses. Then, the obtained bio-fillers were applied to intumescent fire-retardant coatings as synergists, and the effects of shell bio-fillers on the fire resistance and char formation of intumescent fire-retardant coatings were investigated by different analytical methods. TG analysis indicates that the addition of shell bio-fillers can enhance the char-forming ability of the coatings, and the residual weights of IFRC-0, IFRC-CES, IFRC-CHS, and IFRC-CMS at 800 °C are 30.2%, 34.6%, 35.8%, and 37.4%, respectively. The fire protection tests show that the bio-fillers have an excellent synergistic effect on enhancing the fire resistance and char-forming properties of the intumescent fire-retardant coatings, in which CMS exerts the highest synergistic flame-retardant efficiency. The synergistic efficiency of shell bio-fillers in the intumescent coatings varies with the polymorphs of bio-fillers, and aragonite CaCO_3_ shows a better synergistic efficiency than that of calcite CaCO_3_. When the same content of bio-filler is added, CMS consisting of aragonite CaCO_3_ shows the highest synergistic efficiency, CHS composed of the mixture of aragonite CaCO_3_ and calcite CaCO_3_ comes second, and CES composed of calcite CaCO_3_ shows the least effect. Especially, IFRC-CMS coating containing CMS shows the best fire resistance and smoke suppression property, which exhibits a 13.5% reduction in weight loss, 35.8% reduction in char index, 31.1% reduction in flame-spread rating, 32.2% reduction in total heat release, and 29.8% reduction in smoke density rate compared to IFRC-0 coating without bio-filler. Compared to calcite CaCO_3_, aragonite CaCO_3_ has a lower mass loss to coatings, effectively reducing the amount of heat release and smoke generation. The above results show that the aragonite CaCO_3_ of shell bio-fillers is an effective synergist in enhancing the fire resistance and char-forming properties of the intumescent fire-retardant coatings.

## Figures and Tables

**Table 1:** Compositions of the intumescent fire-retardant coatings (mass fraction) %.

Samples	IFR	Bio-Filler	Waterborne Epoxy Resin	Defoamer	Dispersant	Waterborne Epoxy Hardener
IFRC-0	55	0	40	0.5	0.5	4
IFRC-CES	52	3	40	0.5	0.5	4
IFRC-CHS	52	3	40	0.5	0.5	4
IFRC-CMS	52	3	40	0.5	0.5	4

**Table 2:** FTIR assignments for the functional groups of different shell bio-fillers.

FTIR Band (cm^−1^)	Functional Groups	Intensity
3422	–OH stretching [21]	Strong
2923	–CH_2_ stretching [22]	Weak
2519, 2347, 1794	Organics	Strong
1481	Asymmetric stretching of aragonite	Strong
1425	Asymmetric stretching of calcite	Strong
1082	C–O stretching vibration peak of aragonite	Weak
876	CO_3_^2−^ out-of-plane deformation vibration peak of calcite	Strong
859	CO_3_^2−^ out-of-plane deformation vibration peak of aragonite	Strong
713	O–C–O in-plane deformation vibration peak	Strong
700	stretching of aragonite	Weak

**Table 3:** Fire protection performances of IFRCs.

Samples	IFRC-0	IFRC-CES	IFRC-CHS	IFRC-CMS
Weight loss/g	3.7 ± 0.2	3.4 ± 0.1	3.4 ± 0.1	3.2 ± 0.3
Char index/cm^3^	24.3 ± 1.1	19.6 ± 2.6	18.7 ± 0.7	15.6 ± 1.1
Flame-spread rating	20.9 ± 3.3	19.0 ± 1.8	15.0 ± 4.6	14.4 ± 2.4
Intumescent factor	30.0 ± 4.1	38.3 ± 4.7	41.7 ± 2.4	45.0 ± 4.1

**Table 4:** Cone data of the intumescent fire-retardant coatings applied in wood substrate.

Samples	TTI/s	PHRR/(kW·m^−2^)	Time to PHRR/s	THR/(MJ·m^−2^)	Residue/%
IFRC-0	41	94.0	80	5.9	60.0
IFRC-CES	47	87.2	81	4.9	62.1
IFRC-CHS	41	76.3	80	4.3	62.7
IFRC-CMS	44	72.3	79	4.0	64.5

**Table 5:** Thermal data of the IFRCs under nitrogen atmosphere.

Samples	*T*_0_/ °C	*T*_m_/ °C	PMLR/ (%/°C)	Weight Loss/%				*W*_exp_ (800 °C)/%	*W*_theo_ (800 °C)/%	Δ*W* (800 °C)/%
				100~315 °C	315~445 °C	445~600 °C	600~800 °C			
IFRC-0	223.0	362.9	0.6	23.0	36.0	9.3	1.4	30.2	16.8	13.4
IFRC-CES	222.6	363.2	0.6	23.9	30.0	9.4	1.7	34.6	17.6	17.0
IFRC-CHS	223.7	362.0	0.6	25.2	30.1	7.3	1.6	35.8	17.5	18.3
IFRC-CMS	224.1	360.4	0.5	22.8	29.9	8.2	1.7	37.4	17.7	19.7

Notes: *T*_0_, initial decomposition temperature, which was the temperature when the mass loss was up to 5%; PMLR, the peak of mass loss rate; Δ*W* = *W*_exp_ − *W*_theo_; W_exp_ of the waterborne epoxy resin, IFR, CES, CHS, CMS at 800 °C were 6.7%, 25.2%, 50.0%, 52.0%, 55.8%, respectively.

**Table 6:** FTIR assignments for the functional groups of the IFRCs under different treatment temperatures.

FTIR Band (cm^−1^)	Functional Groups	Observations	
		Intensity	Changes
3470, 3420	–NH_2_ stretching	Strong	Disappeared
2956, 2886	–CH_2_ stretching	Strong	Disappeared
1552, 3136	N–H stretching	Strong	Disappeared
1286	P=O stretching	Strong	New, increased
1127	C–O–C stretching	Strong	New, increased
1128	PO_3_^2−^ stretching	Strong	Disappeared
1045, 1017, 944,	P–O–C stretching	Strong	New, increased
1016	C–O stretching	Strong	Disappeared
758, 738	C–H deformation for benzene ring	Strong	New, increased
674, 874	P–O–P stretching	Strong	Disappeared

## Data Availability

The data presented in this study are available upon request from the corresponding author.
